# Intracerebral hemorrhage outcomes following selective blockade or stimulation of the PGE_2_ EP1 receptor

**DOI:** 10.1186/s12868-015-0182-2

**Published:** 2015-08-01

**Authors:** Jenna L Leclerc, Abdullah S Ahmad, Nilendra Singh, Luke Soshnik-Schierling, Ellis Greene, Alex Dang, Sylvain Doré

**Affiliations:** Department of Anesthesiology, University of Florida, Gainesville, FL USA; Department of Neuroscience, University of Florida, Gainesville, FL USA; Departments of Neurology, Psychiatry, Psychology and Pharmaceutics, University of Florida, Gainesville, FL USA; University of Florida College of Medicine, 1275 Center Drive, Gainesville, FL 32610-0159 USA

**Keywords:** 17-pt-PGE_2_, EP3, Gliosis, Iron, Neuroinflammation, Neuroprotection, PECAM, SC-51089, Stroke

## Abstract

**Background:**

Inflammation following intracerebral hemorrhage (ICH) significantly contributes to secondary brain damage and poor outcomes. Prostaglandin E_2_ (PGE_2_) is known to modulate neuroinflammatory responses and is upregulated in response to brain injury as a result of changes in inducible cyclooxygenase 2 (COX-2) and the membrane-bound type of PGE synthase. Inhibition of COX-2 activity has been reported to attenuate ICH-induced brain injury; however, the clinical utility of such drugs is limited due to the potential for severe side effects. Therefore, it is now important to search for downstream targets capable of preferentially modulating PGE_2_ signaling, and the four E prostanoid receptors, EP1-4, which are the main targets of PGE_2_, remain a viable therapeutic option. We have previously shown that EP1 receptor deletion aggravates ICH-induced brain injury and impairs functional recovery, thus the current study aimed to elaborate on these results by including a pharmacologic approach targeting the EP1 receptor.

**Results:**

Chronic post-treatment with the selective EP1 receptor antagonist, SC-51089, increased lesion volume by 30.1 ± 14.5% (p < 0.05) and treatment with the EP1 agonist, 17-pt-PGE_2_, improved neuromuscular functional recovery on grip strength (p < 0.01) and hanging wire (p < 0.05) behavioral testing. To begin identifying the mechanisms involved in EP1-mediated neuroprotection after ICH, histology was performed to assess ferric iron content, neuroinflammation, leukocyte transendothelial migratory potential, and peripheral neutrophil and immunoglobulin infiltration. Following ICH, mice treated with the antagonist displayed increased ferric iron (p < 0.05) and cortical microgliosis (p < 0.05), whereas treatment with the agonist decreased cortical (p < 0.01) and striatal (p < 0.001) astrogliosis, leukocyte transendothelial migratory potential (p < 0.01), neutrophil infiltration (p < 0.05), and blood brain barrier breakdown (p < 0.05).

**Conclusions:**

In agreement with our previous results, selective antagonism of the EP1 receptor aggravated ICH-induced brain injury. Furthermore, EP1 receptor agonism improved anatomical outcomes and functional recovery. Thus, the present data continues to reinforce a putative role for EP1 as a new and more selective therapeutic target for the treatment of ICH that could reduce the side effects associated with COX-2 inhibition while still exploiting the beneficial effects.

## Background

Intracerebral hemorrhage (ICH) is the most debilitating and is associated with the highest mortality rates of all stroke subtypes, has no effective therapeutics, and represents a major public health problem [1, 2]. ICH is a multifaceted disorder with acute and chronic mechanisms of injury, including initial mechanical damage due to the expanding hematoma and compression of brain tissue and later inflammatory/oxidative processes primarily resulting from the presence of blood components [3–5]. Combating the strong neuroinflammatory cascade after ICH remains a promising therapeutic target to reduce secondary brain damage and improve patient outcomes [4, 6, 7]. Neuroinflammation following brain injury is intimately connected to upregulation of cyclooxygenase (COX) enzymes, mainly the inducible isoform COX-2, coordinated expression of membrane-bound PGE synthase-1 (mPGES-1), and consequent increased production of prostaglandins (e.g. PGE_2_) [8]. Prostaglandins have been shown to modulate outcomes in a variety of neurological disorders, including stroke, traumatic brain injury, epilepsy, Alzheimer’s disease (AD), and Parkinson’s disease (PD), among others [9–16]. Although selective inhibition of COX-2 has been reported to improve outcomes in many preclinical models of these neurological disorders, including ICH, the clinical use of these drugs is limited due to the potential for known severe side effects [17–21]. Therefore, targeting more specific downstream pathways of COX/mPGES-mediated prostaglandin signaling may reduce these deleterious side effects while still harnessing the already characterized positive immunomodulatory properties seen with COX-2 inhibition.

Prostaglandin E_2_ (PGE_2_) is a major product of COX/PGES activity in the central nervous system and is classically thought of as proinflammatory within the brain [16, 22, 23]. However, emerging evidence suggests a complex scenario with PGE_2_ capable of exerting pro- and/or anti-inflammatory functions depending on the underlying neuropathophysiology [9, 11, 22, 24]. The many actions of PGE_2_ are produced by its differential stimulation of mainly the G-protein-coupled E prostanoid (EP) receptors 1-4, which display varied anatomical distributions, cellular expression profiles, ligand binding affinity, desensitization kinetics, and signal transduction pathways [25–27]. In the present study, we focus on the EP1 receptor, which is reported to be expressed in the thalamus/hypothalamus, hippocampus, cortex, striatum, and cerebellum [22]. EP1 stimulation results in increased intracellular calcium levels via coupling to a Gαq protein, phosphatidyl inositol hydrolysis, and extracellular calcium influx [28, 29].

We and others have documented the pro- and/or anti-inflammatory effects and neurotoxic or neuroprotective properties of signaling through the PGE_2_-EP1 axis in various models of acute and chronic neurological disorders such as stroke, hypoxic-ischemic encephalopathy (HIE), traumatic brain injury (TBI), AD, and PD [10, 11, 14, 15, 24, 28, 30–32]. In models of transient forebrain ischemia, focal ischemia, and excitotoxicity, PGE_2_-EP1 signaling aggravates brain damage possibly by increasing blood brain barrier (BBB) disruption and vascular tone and decreasing neuronal resistance to oxidative stress and cell death [24, 28, 30, 31]. Similarly, the neurotoxicity associated with PGE_2_-EP1 signaling was shown in a combined model of AD and cerebral ischemia, where amyloid plaques and neuronal damage were reduced in EP1-deleted AD mice following ischemic insult [15]. Further, inhibition of EP1 with a selective antagonist reduced HIE cerebral injury [32]. In a model of toxin-induced Parkinsonism, dopaminergic neurons were protected and apomorphine-induced contralateral rotations were decreased in EP1^−/−^ mice [14]. On the contrary, deletion or antagonism of the EP1 receptor failed to provide neuroprotection in a contusive model of TBI [10]. Last, we have recently shown that genetic deletion of the EP1 receptor aggravates ICH-induced brain injury and impairs functional recovery, where mechanistic evidence was provided to suggest EP1 stimulation positively modulates microglial activation and phagocytosis [11]. Thus, signaling through the PGE_2_-EP1 axis has a complex role following brain injury and appears to depend on the underlying pathologic processes, and possibly also on the genetic and/or pharmacologic approach used.

The present study aimed to explore our prior data in EP1^−/−^ mice by extension to post-ICH pharmacologic manipulation of the EP1 receptor. In line with our previous results, here we show that selective antagonism of the EP1 receptor with SC-51089 chronically following ICH exacerbates brain injury and increases ferric iron deposition and microgliosis. Furthermore, repeated administration of 17-pt-PGE_2_, an EP1 agonist, after ICH improved functional recovery and reduced astrogliosis, leukocyte transendothelial migratory potential, peripheral neutrophil infiltration, and BBB breakdown.

## Methods

### Mice

Experiments were performed with 11–13-week-old C57BL/6 male mice. The colony was bred and maintained in our animal facilities in a temperature-controlled reverse light cycle environment (23 ± 2°C, 12-h light/dark cycle) so that behavioral testing could be performed during the awaken phase. Mice were allowed free access to food and water before and after surgical procedures. All animal protocols were approved by the University of Florida Institutional Animal Care and Use Committee.

### ICH model

ICH was induced by unilateral intrastriatial infusion of collagenase type VII-S (Sigma, St. Louis, MO, USA) as we have described previously [11]. Briefly, mice were anesthetized with isoflurane (3% induction, 1–1.5% maintenance) and immobilized on a stereotactic frame (Stoelting, Wood Dale, IL, USA). After making a small incision in the skin overlying the skull, a craniotomy was performed 0.5 mm anterior and 2.4 mm right of bregma. Collagenase (0.04 units in 0.2 µl of sterile saline) was infused 3.2 mm ventral from the dura over a 5-min period using a syringe with a 26-gauge blunt tip needle (Hamilton Co., Reno, NV, USA). Rectal temperatures were maintained at 37.0 ± 0.5°C throughout all surgical procedures and mice were allowed to recover in temperature- and humidity-controlled chambers for at least 1 h postoperatively.

### Experimental groups and drug treatments

Mice (n = 30) were equally distributed into three treatment groups: antagonist, agonist, and control. The antagonist 8-chloro-2-[1-oxo-3-(4-pyridinyl)propyl]hydrazide-dibenz[b,f][1,4]oxazepine-10(11H)-carboxylic acid, monohydrochloride] (SC-51089) is selective for the EP1 receptor (K_i_ = 1260 nM) and was administered at a dose of 10 µg/kg of body weight. Intraperitoneal treatment with this dose either 5 min or 6 h after reperfusion significantly reduced infarct volume and brain swelling and improved neurological deficits in the transient middle cerebral artery occlusion stroke model [33]. The agonist 9-oxo-11α,15S-dihydroxy-17-phenyl-18,19,20-trinor-prosta-5Z,13E-dien-1-oic acid (17-pt-PGE_2_) is non-selective for the EP1 receptor, with K_i_ values of 12.6 and 3.7 nM for the EP1 and EP3 receptors, respectively [34]. The dose of 0.3 mg/kg of body weight was chosen based on a previous study where intravenous pretreatment (10 min prior) was shown to aggravate histamine-mediated gastric mucosal injury [35]. SC-51089 and 17-pt-PGE_2_ were purchased from Cayman Chemicals (Ann Arbor, MI, USA). Stock solutions were prepared in dimethyl sulfoxide (DMSO) and subsequently aliquoted and stored at −20°C. Solutions for injections were freshly diluted from stock aliquots in sterile saline such that the final concentration of DMSO was only 0.001% and mice were appropriately rehydrated. The control group received an equivalent volume saline injection. Injections were performed subcutaneously immediately following surgical procedures, 6 h post-hemorrhage, and at 12-h intervals thereafter until the 72-h endpoint.

### Neurobehavioral testing

Functional outcomes were assessed at 24, 48, and 72 h after ICH with the following neurobehavioral tests: grip strength, hanging wire task, accelerating rotarod performance, and open field locomotor activity. For all tests, baseline function was assessed the day prior to surgery. Testing was performed during the dark cycle (awaken phase) by investigators blinded to treatment group. Each test was performed at the same time of day and mice were allowed 30–45 min of rest between tests. Grip strength: forelimb strength was measured using the Animal Grip Strength System (San Diego Instruments, San Diego, CA, USA). Mice were suspended by the tail over the testing grid until their forepaws had a grip on the steel bar and then pulled away from the grid until they released their grip. Five consecutive trials per mouse were performed for each testing period. Data are reported as the average maximal force recorded prior to the mouse releasing its hold on the bar. Hanging wire task: mice were allowed to grip a steel wire suspended 50 cm above a padded flat surface with their two forelimbs and the latency to fall was recorded to evaluate muscle strength and condition. Accelerating rotarod performance: mice were assessed for motor deficits and coordination, endurance, and balance using an accelerating rotarod Rotamex-5 machine and software (Columbus, OH, USA). Mice were trained on the three consecutive days prior to surgery, where the last training period served as baseline functioning. Rotational speed started at 5 rpm and ended at 50 rpm and the latency to fall was automatically collected by the Rotamex-5 software. Open field locomotor activity: ambulatory time was measured using an automated open field activity monitor and video tracking interface system (MED Associates, St. Albans, VT, USA). Briefly, mice were placed individually in four transparent acrylic cages and their activity was recorded over a 20-min test period. Data are represented as ambulatory counts omitting the first 5 min to exclude for initial anxiety responses.

### Histological procedures and quantification

At 72 h post-ICH, mice were deeply anesthetized and transcardially perfused with phosphate-buffered saline (PBS, pH 7.4) followed by 4% paraformaldehyde (PFA). Brains were collected and stored in 4% PFA for at least 24 h prior to cryopreservation in a 30% sucrose/PBS solution. Sections were processed at 30 µm using a Leica CM 1850 cryostat (Buffalo Grove, IL, USA) and stored at −80°C for later histological procedures. Cresyl violet staining was used for determination of lesion volume [24]. To assess ferric iron content, Perl’s iron staining was completed by incubating slides in a 1:1 mix of 2% hydrochloric acid and 2% potassium ferrocyanide for 20 min, followed by counterstaining with nuclear fast red. Immunohistochemistry was performed to evaluate microglial activation, astrogliosis, leukocyte transendothelial migratory potential, neutrophil infiltration, and BBB breakdown using the following primary antibodies: ionized calcium-binding adapter protein 1 (Iba1), 1:1000 (Wako, Richmond, VA, USA); glial fibrillary acidic protein (GFAP), 1:1000 (Dako, Carpinteria, CA); platelet endothelial cell adhesion molecule 1 (PECAM-1), 1:400 (Santa Cruz, Dallas, TX, USA); myeloperoxidase (MPO), 1:500 (Pierce, Dallas, TX, USA); and immunoglobulin G (IgG), 1:300 (Vector Laboratories, Burlingame, CA, USA), respectively. A secondary biotinylated antibody was used for detection (Vector Laboratories), except for IgG staining, which used a biotinylated primary anti-mouse IgG antibody. The Vectastain Elite ABC kit and DAB kit (Vector Laboratories) were used per manufacturer’s instructions for the avidin-peroxidase step and final DAB reaction, respectively. GFAP and Iba1 slides were not counterstained, MPO and IgG slides were counterstained with Cresyl violet, and PECAM-1 slides were counterstained with nuclear fast red. After Cresyl violet, Perl’s iron, and immunohistochemical staining, slides were dehydrated in increasing concentrations of ethanol and coverslipped with Permount.

All slides were scanned using a ScanScope CS and analyzed with ImageScope software (Aperio Technologies, Inc., Vista, CA, USA). Lesion volume was determined by outlining of the injured areas on 32 sections equally distributed throughout the entire hematoma and anteroposterior brain regions. Lesion areas for each section were abstracted from the ImageScope software and a lesion volume was calculated using these areas, known distance between each section, and section thickness. Ferric iron content and immunohistochemical stains were quantified using the ImageScope ‘Positive Pixel Count’ algorithm after the appropriate brain regions were outlined (see below). Algorithms were tuned for each of the stains such that the appropriate signal and strength of signal was detected. As an example, moderate and strongly positive blue pixels are detected in Perl’s iron slides, and not weakly positive pixels because these could potentially represent non-specific signal. For each stain, the same four sections representing maximal lesion areas were used. Cortical microgliosis was analyzed by placing identically sized boxes of 1,500 by 1,500 pixels in the ipsilateral and contralateral cortex. Data are presented as the relative ipsilateral to contralateral signal. Striatal microgliosis was analyzed by outlining of the ipsilateral and contralateral striatum, excluding the lesion area. Data are presented as the ipsilateral signal per area quantified with normalization for the contralateral signal per area quantified. Cortical and striatal astrogliosis were analyzed in a similar manner to microgliosis; however, these data were not normalized due to negligible signal in the contralateral cortex and striatum with the thresholds used here. Cortical and striatal astrogliosis data are presented as the signal per area quantified. Perl’s, MPO, and PECAM-1 slides were analyzed by circling of the ipsilateral hemisphere. Whole brain signal was examined for IgG quantification because staining extended into the contralateral side in some cases. After all analyses, the appropriate algorithm was run and data was abstracted from the ImageScope software.

### Statistical analyses

GraphPad Prism 6 software was used for all statistical analyses (San Diego, CA, USA). Differences between two groups were determined by an unpaired two-tailed parametric Student’s *t* test. Data are expressed as mean ± SEM, and p < 0.05 was considered statistically significant in all comparisons.

## Results

Mice were treated after ICH with the selective EP1 receptor antagonist SC-51089 or the EP1/EP3 receptor agonist 17-pt-PGE_2_ and various anatomical and functional outcomes were evaluated by histological staining and neurobehavioral testing, respectively. Treatment with the antagonist resulted in a 20% mortality rate, whereas no mortality was seen in the agonist or control groups.

### Effect of blockade or stimulation of the EP1 receptor on ICH-induced brain injury

**Fig. 1:**
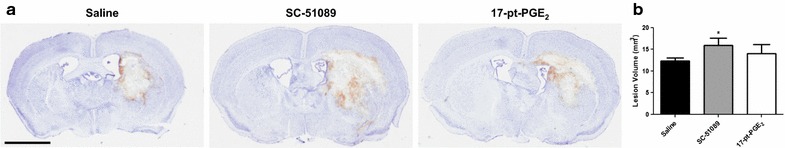
Effect of blockade or stimulation of the EP1 receptor on ICH-induced brain injury. Antagonist (SC-51089, 10 µg/kg), agonist (17-pt-PGE_2_, 0.3 mg/kg), or vehicle (saline) was administered subcutaneously at the onset of injury, 6 h post-ICH, and at 12-h intervals thereafter. Seventy-two hours after ICH, brains were harvested and sections processed for Cresyl violet staining and lesion volume determination. **a** Representative photomicrographs of coronal brain sections from control (*left panel*), SC-51089- (*middle panel*), and 17-pt-PGE_2_- (*right panel*) treated mice. *Scale bar* 1 mm. **b** Quantification showed that SC-51089-treated mice had significantly more ICH-induced brain injury, whereas no significant differences were seen with 17-pt-PGE_2_ treatment. *p < 0.05 when compared to the control group, n = 8–10 per group.

Striatal hemorrhages were reproducible in all treatment groups (Fig. 1a). Quantification of lesion volumes showed that SC-51089-treated animals had 30.1 ± 14.5% larger lesions when compared to the control group (15.92 ± 1.67 vs. 12.35 ± 0.72 mm^3^, p < 0.05; Fig. 1b), whereas no significant differences were seen for the 17-pt-PGE_2_-treated mice.

### Effect of blockade or stimulation of the EP1 receptor on functional outcomes

**Fig. 2:**
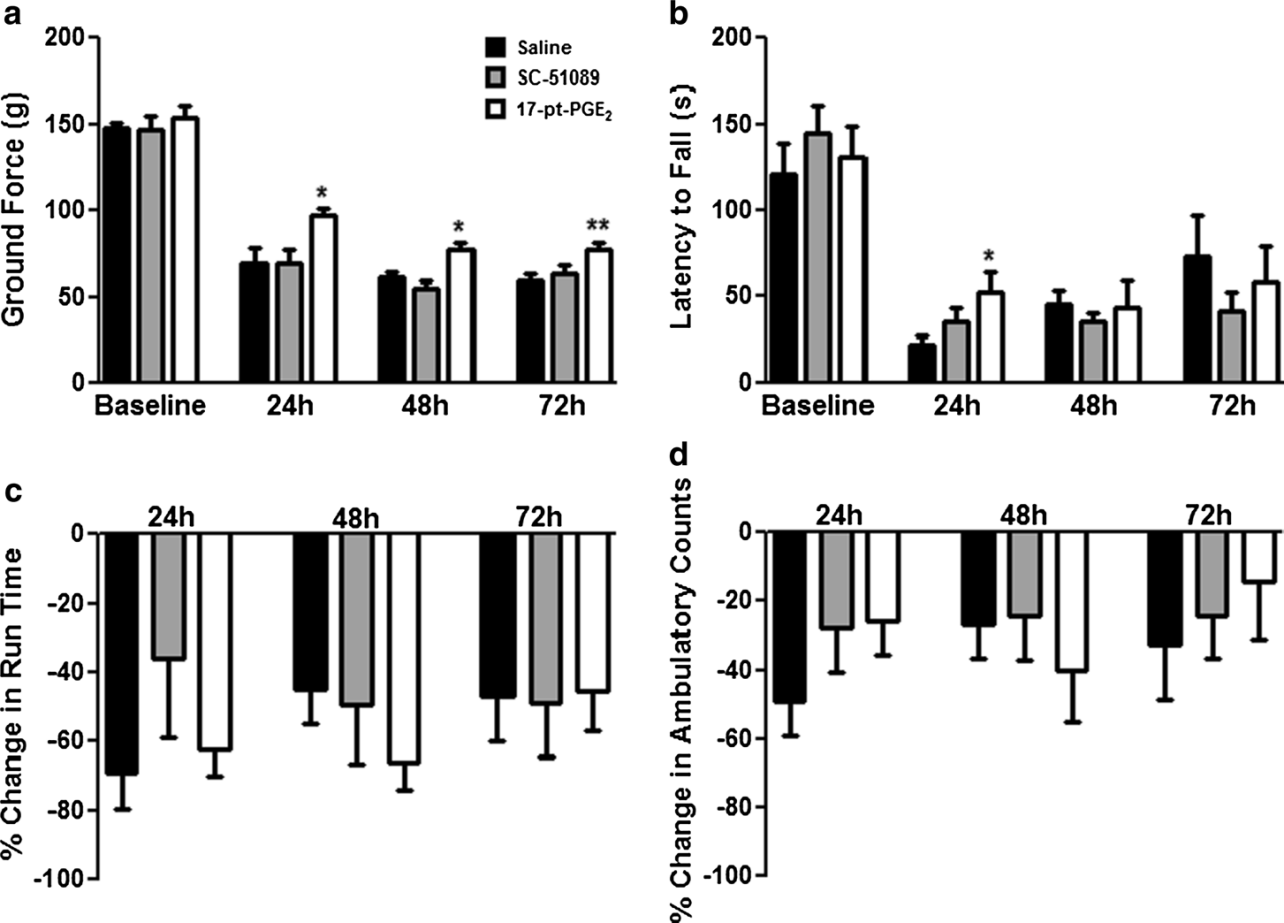
Effect of blockade or stimulation of the EP1 receptor on functional outcomes after ICH. Antagonist (SC-51089, 10 µg/kg), agonist (17-pt-PGE_2_, 0.3 mg/kg), or vehicle (saline) was administered subcutaneously at the onset of injury, 6 h post-ICH, and at 12-h intervals thereafter. Neurobehavioral testing was performed at 24, 48, and 72 h after ICH by individuals blinded to the treatment groups. No significant differences in baseline functioning were seen between the treatment groups on any of the neurobehavioral tests. **a** Grip strength testing showed that mice treated with 17-pt-PGE_2_ had significantly improved forelimb strength at all testing time points post-ICH, whereas no significant differences were seen with SC-51089 treatment. **b** Mice treated with 17-pt-PGE_2_ had significantly improved latency to fall on the hanging wire task at 24 h following ICH, whereas no significant differences were seen with SC-51089 treatment. **c** Mice in all treatment groups displayed similar performance on an accelerating rotarod after ICH. **d** Treatment with SC-51089 or 17-pt-PGE_2_ did not affect post-ICH ambulatory activity. *p < 0.05 and **p < 0.01 when compared to the control group, n = 8–10 per group.

Neurobehavioral testing was performed daily following ICH by investigators blinded to treatment group. All mice within the study participated in all behavioral testing and no significant differences in baseline functioning were seen between the treatment groups on any of the behavioral tests employed here. The 17-pt-PGE_2_-treated mice exhibited improved neurologic function when compared to the control group on two of the four behavioral tests performed, whereas no significant differences in functional recovery were seen for the SC-51089-treated mice (Fig. 2). Grip strength: The average baseline grip strength for the control and 17-pt-PGE_2_ groups were 147.9 ± 3.5 and 154.4 ± 6.9 g, respectively, and these values were not statistically different. At all testing periods post-ICH, all treatment groups demonstrated significantly impaired grip strength when compared to baseline testing (p < 0.0001). However, at 24 h post-ICH, 17-pt-PGE_2_-treated mice had significantly improved forelimb strength when compared to the control group (97.2 ± 4.0 vs. 69.2 ± 9.3 g, p < 0.05; Fig. 2a). These mice continued to have better forelimb muscular function at 48 h (76.7 ± 4.5 vs. 61.1 ± 3.4 g, p < 0.05; Fig. 2a) and 72 h (77.5 ± 4.2 vs. 59.6 ± 4.0 g, p < 0.01; Fig. 2a) post-ICH. Hanging wire task: mice treated with 17-pt-PGE_2_ were able to hang for longer times on a suspended wire when compared to the control group at 24 h after ICH (52.2 ± 12.1 vs. 22.3 ± 4.7 s, p < 0.05; Fig. 2b). Average baseline latency to fall from the wire was not significantly different between the two groups (17-pt-PGE_2_: 131.3 ± 17.7 s, control: 121.1 ± 18.5 s). There were no significant differences in performance on an accelerating rotarod (Fig. 2c) or open field locomotor activity (Fig. 2d) for the 17-pt-PGE_2_-treated group. For the SC-51089-treated mice, no significant differences in functional outcomes were seen at any time point post-ICH with the behavioral tests used in this study.

### Effect of blockade or stimulation of the EP1 receptor on brain ferric iron content

**Fig. 3:**
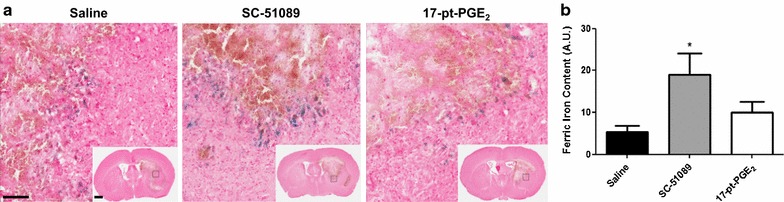
Brain ferric iron content following ICH and blockade or stimulation of the EP1 receptor. Antagonist (SC-51089, 10 µg/kg), agonist (17-pt-PGE_2_, 0.3 mg/kg), or vehicle (saline) was administered subcutaneously at the onset of injury, 6 h post-ICH, and at 12-h intervals thereafter. Seventy-two hours after ICH, brains were harvested and sections processed for Perl’s staining and ferric iron content determination. **a** Representative high magnification photomicrographs showing ferric iron (*blue*) accumulation in the perihematomal regions of control (*left panel*), SC-51089- (*middle panel*), and 17-pt-PGE_2_- (*right panel*) treated mice. *Square* selections in the insets denote magnified regions. *Scale bars* on the magnified images and insets are 100 µm and 1 mm, respectively. **b** Quantification of brain ferric iron content in the ipsilateral hemisphere showed that SC-51089-treated mice had significantly more ferric iron accumulation, whereas no significant differences were seen for the 17-pt-PGE_2_-treated mice. No ferric iron deposition was seen in the contralateral hemisphere for any of the mice in the study. *p < 0.05 when compared to the control group, n = 8–10 per group.

To begin analyzing the mechanisms involved in EP1-mediated neuroprotection following ICH, Perl’s iron staining was performed to assess brain ferric iron content. Ferric iron predominately accumulated in perihematomal regions in all treatment groups (Fig. 3a). Quantification revealed that SC-51089-treated animals had 251.3 ± 93.4% more ferric iron in the ipsilateral hemisphere when compared to the control group (18.99 ± 5.05 vs. 5.41 ± 1.48 A.U., p < 0.05; Fig. 3b), whereas no significant differences were seen for the 17-pt-PGE_2_-treated mice. No ferric iron deposition was seen in the contralateral hemisphere for any of the mice in the study.

### Effect of blockade or stimulation of the EP1 receptor on microglial activation and astrogliosis

**Fig. 4:**
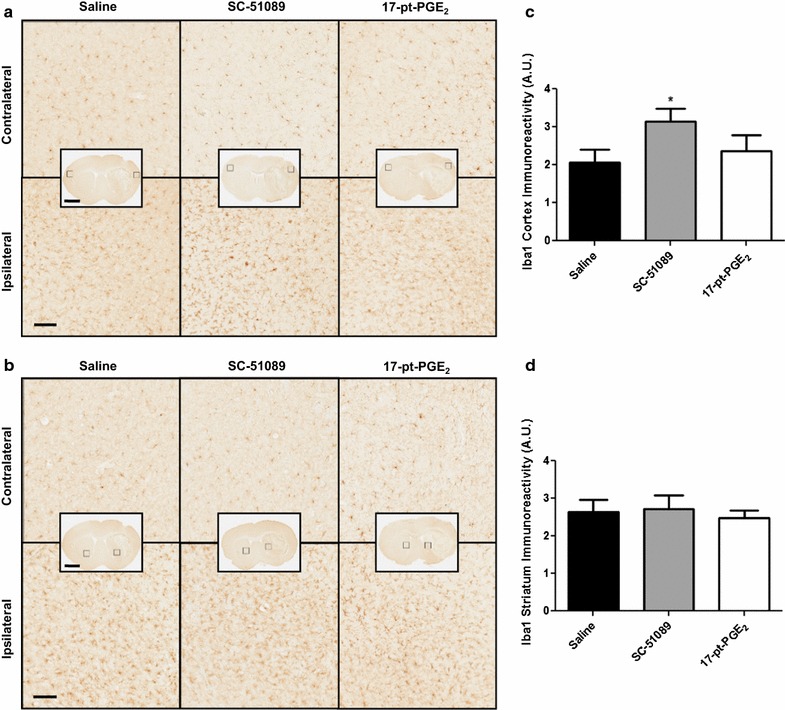
Iba1 expression following ICH and blockade or stimulation of the EP1 receptor. Antagonist (SC-51089, 10 µg/kg), agonist (17-pt-PGE_2_, 0.3 mg/kg), or vehicle (saline) was administered subcutaneously at the onset of injury, 6 h post-ICH, and at 12-h intervals thereafter. Seventy-two hours after ICH, brains were harvested and sections processed for Iba1 immunohistochemistry to evaluate cortical and striatal microgliosis. **a**, **b** Representative high magnification photomicrographs showing the ipsilateral and contralateral **a** cortex and **b** striatum for Iba1 immunohistochemistry of coronal brain sections from control (*left panels*), SC-51089- (*middle panels*), and 17-pt-PGE_2_- (*right panels*) treated mice. *Square* selections in the insets denote magnified regions. *Scale bars* on the magnified images and insets are 100 µm and 2 mm, respectively. **c**, **d** Quantification of Iba1 immunoreactivity demonstrated that SC-51089-treated mice had significantly more **c** cortical microgliosis, whereas no significant differences were seen in (**d**) striatal microgliosis. This increased cortical Iba1 immunoreactivity was accompanied by more cellular morphological changes. No significant differences in **c** cortical or **d** striatal microgliosis were seen for the 17-pt-PGE_2_-treated mice. Data is normalized to the corresponding contralateral equivalent signal with appropriate control for the area of quantification in striatal analyses (see “Methods”). *p < 0.05 when compared to the control group, n = 8–10 per group.

To further delineate mechanisms, cortical and striatal microglial activation and astrogliosis were identified by Iba1 and GFAP immunohistochemistry, respectively. Following ICH, SC-51089-treated mice displayed more cortical microglial activation and morphological changes when compared to the control group, whereas no significant differences were seen in striatal microgliosis or for the 17-pt-PGE_2_-treated mice. All treatment groups displayed increased cortical (Fig. 4a) and striatal (Fig. 4b) microglial activation and morphological changes in the ipsilateral hemisphere relative to the contralateral equivalent areas. Quantification of cortical Iba1 immunoreactivity showed that SC-51089-treated mice had 52.8 ± 16.7% more microgliosis when compared to the control group (3.14 ± 0.34 vs. 2.05 ± 0.34 A.U., p < 0.05; Fig. 4c). Cortical microgliosis was not significantly different for the 17-pt-PGE_2_-treated group (17-pt-PGE_2_: 2.37 ± 0.41 A.U., control: 2.05 ± 0.34 A.U.; Fig. 4c). No significant differences in striatal microgliosis were seen for either treatment group (SC-51089: 2.73 ± 0.34 A.U., 17-pt-PGE_2_: 2.48 ± 0.20 A.U., control: 2.65 ± 0.31 A.U.; Fig. 4d).

**Fig. 5:**
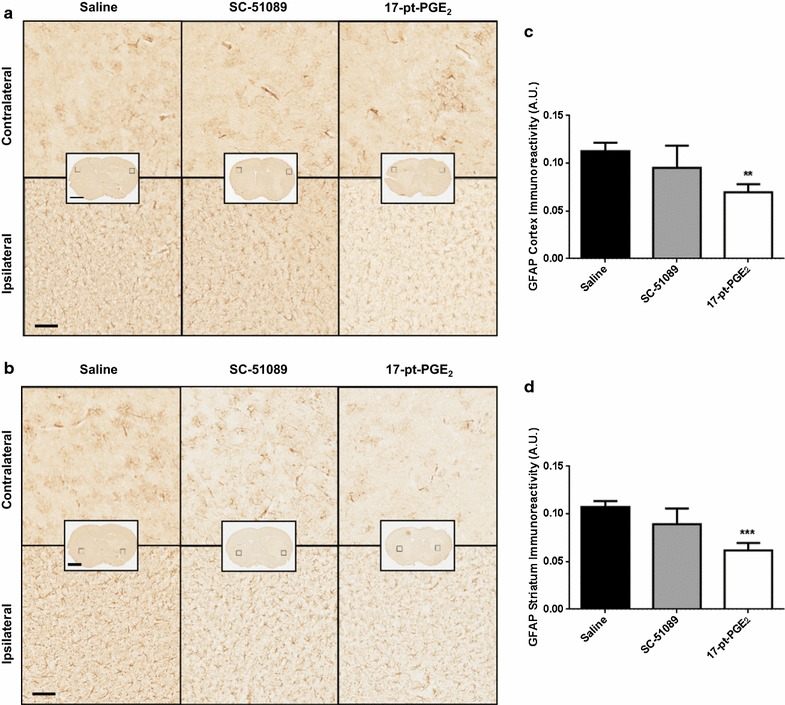
GFAP expression following ICH and blockade or stimulation of the EP1 receptor. Antagonist (SC-51089, 10 µg/kg), agonist (17-pt-PGE_2_, 0.3 mg/kg), or no treatment (saline) was administered subcutaneously at the onset of injury, 6 h post-ICH, and at 12-h intervals thereafter. Seventy-two hours after ICH, brains were harvested and sections processed for GFAP immunohistochemistry to evaluate cortical and striatal astrogliosis. **a**, **b** Representative high magnification photomicrographs showing the ipsilateral and contralateral **a** cortex and **b** striatum for GFAP immunohistochemistry of coronal brain sections from control (*left panels*), SC-51089- (*middle panels*), and 17-pt-PGE_2_- (*right panels*) treated mice. *Square* selections in the insets denote magnified regions. *Scale bars* on the magnified images and inserts are 100 µm and 2 mm, respectively. **c, d** Quantification of GFAP immunoreactivity demonstrated that 17-pt-PGE_2_-treated mice had significantly less **c** cortical and **d** striatal astrogliosis, whereas no significant differences were seen for SC-51089-treated mice. All treatment groups demonstrated negligible staining in the contralateral cortex and striatum; thus, data is presented as the ipsilateral GFAP immunoreactivity corrected for the area of quantification, without normalization for the contralateral equivalent areas. **p < 0.01 and ***p < 0.001 when compared to the control group, n = 8–10 per group.

When compared to the control group, 17-pt-PGE_2_-treated mice had significantly less cortical and striatal astrogliosis, whereas no significant differences were seen with SC-51089 treatment. Similar to microgliosis, all treatment groups displayed significantly increased ipsilateral cortical (Fig. 5a) and striatal (Fig. 5b) astrogliosis compared to the contralateral equivalent areas. Quantification of GFAP immunoreactivity showed that 17-pt-PGE_2_-treated mice had 38.1 ± 7.7% less cortical astrogliosis (0.0697 ± 0.0086 A.U. vs. 0.1126 ± 0.0089, p < 0.01; Fig. 5c) and 42.6 ± 7.3% less striatal astrogliosis (0.0616 ± 0.0079 A.U. vs. 0.1072 ± 0.0062, p < 0.001; Fig. 5d). No significant differences in cortical (SC-51089: 0.0951 ± 0.0232 A.U., control: 0.1126 ± 0.0089 A.U.; Fig. 5c) or striatal (SC-51089: 0.0892 ± 0.0164 A.U., control: 0.1072 ± 0.0062 A.U.; Fig. 5d) astrogliosis were seen for the SC-51089-treated group.

### Effect of blockade or stimulation of the EP1 receptor on leukocyte transendothelial migratory potential, peripheral neutrophil infiltration, and BBB breakdown

**Fig. 6:**
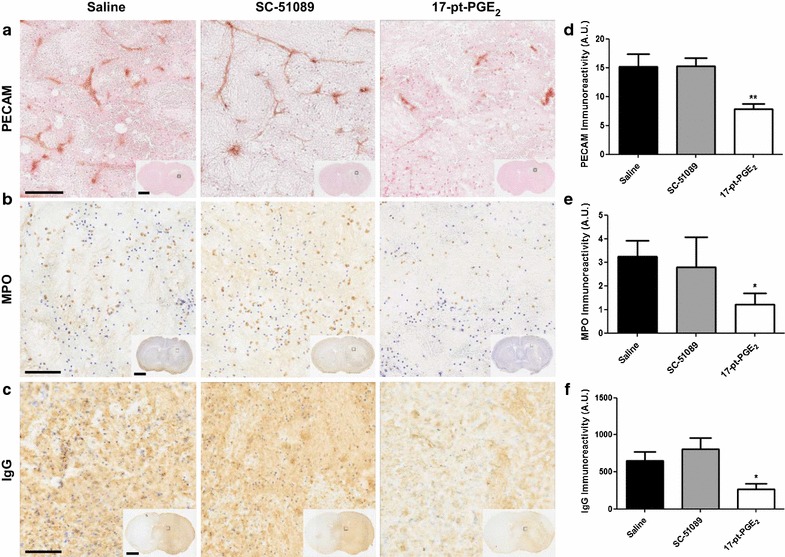
PECAM-1, MPO, and IgG immunoreactivity following ICH and blockade or stimulation of the EP1 receptor. Antagonist (SC-51089, 10 µg/kg), agonist (17-pt-PGE_2_, 0.3 mg/kg), or vehicle (saline) was administered subcutaneously at the onset of injury, 6 h post-ICH, and at 12-h intervals thereafter. Seventy-two hours after ICH, brains were harvested and sections processed for PECAM-1, MPO, and IgG immunohistochemistry to evaluate leukocyte transendothelial migratory potential, neutrophil infiltration, and BBB breakdown, respectively. **a**, **b**, **c** Representative high magnification photomicrographs showing **a** PECAM-1 staining highlighting blood vessels within the lesion, **b** MPO-positive neutrophils within the lesion, and **c** IgG staining within the lesion and surrounding areas of coronal brain sections from control (*left panels*), SC-51089- (*middle panels*), and 17-pt-PGE_2_- (*right panels*) treated mice. *Square* selections in the insets denote magnified regions. *Scale bars* on the magnified images and inserts are 100 µm and 2 mm, respectively. **d**, **e**, **f** Quantification revealed that 17-pt-PGE_2_-treated mice had significantly less **d** PECAM-1 staining, **e** neutrophil infiltration, and **f** BBB breakdown. All quantification data are presented at the same scale such that the relative **d** PECAM-1, **e** MPO, and **f** IgG immunoreactivity can be directly compared. No significant differences were seen for the SC-51089-treated mice. *p < 0.05 and **p < 0.01 when compared to the control group, n = 7–10 per group.

Additional immunohistochemical staining for PECAM-1, MPO, and IgG was performed to identify leukocyte transendothelial migratory potential, peripheral neutrophil infiltration, and BBB breakdown, respectively. PECAM-1 (Fig. 6a) and MPO (Fig. 6b) staining was only observed within injured brain regions. IgG staining was diffusely present throughout the ipsilateral hemisphere and crossed into the contralateral hemisphere in some cases (Fig. 6c). Quantification showed that 17-pt-PGE_2_-treated mice had 48.4 ± 6.5% less PECAM-1 immunoreactivity when compared to the control group (7.83 ± 0.98 vs. 15.17 ± 2.18 A.U., p < 0.01; Fig. 6d). Likewise, these mice also had 62.6 ± 14.7% less neutrophil infiltration (1.21 ± 0.48 vs. 3.24 ± 0.67 A.U., p < 0.05; Fig. 6e) and 59.1 ± 11.9% less IgG immunoreactivity (0.265 ± 0.077 A.U. vs. 0.649 ± 0.121 A.U., p < 0.05; Fig. 6f). No significant differences in PECAM-1, MPO, or IgG immunoreactivity were observed for the SC-51089-treated group.

## Discussion

We have shown that chronic post-treatment with SC-51089, a selective EP1 receptor antagonist, aggravates brain injury after ICH, resulting in larger lesion volumes and more iron accumulation, whereas treatment with 17-pt-PGE_2_, an EP1 receptor agonist with extensive EP3 receptor agonism, results in no detectable changes in lesion volume or iron deposition, but improves neuromuscular functional recovery. The two treatments resulted in differential patterns of microglial and astrocyte activation, with EP1 antagonism producing increased cortical microgliosis and EP1/EP3 agonism (later referred to as EP1 agonism or EP1 agonist) resulting in reduced cortical and striatal astrogliosis. Last, treatment with the EP1 agonist reduced leukocyte transendothelial migratory potential, neutrophil infiltration, and BBB breakdown.

Our results demonstrating that post-ICH EP1 antagonism exacerbates brain injury are in agreement with our previous study that showed significantly more ICH-induced brain injury and worse functional outcomes in mice with genetic deletion of the EP1 receptor [11], although we did not observe smaller lesion volumes with the EP1 agonist used in this study, 17-pt-PGE_2_. This EP1 agonist has K_i_ values of 12.6 and 3.7 nM for the EP1 and EP3 receptors [34], respectively, and the non-selective nature could have led to the outcomes seen here. In support of this hypothesis, we have recently shown that EP3^−/−^ mice have less ICH-induced brain injury when compared to WT controls, implying an injurious role for PGE_2_-EP3 signaling that is opposite to the neuroprotective effects of the EP1 receptor [36]. Therefore, it is likely that the overall desired beneficial effect of 17-pt-PGE_2_ by agonism of the EP1 receptor is negated by simultaneous EP3 receptor agonism, resulting in no observed differences with 17-pt-PGE_2_ treatment when compared to the control group. It is also possible that the dose used here for agonist treatment (selected based on previous systemic studies and not in the context of stroke) may not be high enough to affect lesion volume after ICH. Interestingly, the 17-pt-PGE_2_-treated mice performed better on neurobehavioral testing, including grip strength examination and the hanging wire task. In our aforementioned observations with EP3^−/−^ mice, we did not find the same extent of differences on neurobehavioral testing as compared to EP1^−/−^ mice, and thus collectively these data imply an important role for the PGE_2_-EP1 signaling axis in post-ICH functional recovery. Additional studies with 17-pt-PGE_2_ treatment in the appropriate EP receptor knockout animals would be necessary to verify this suggestion. Furthermore, studies in other ICH models and using additional selective EP1 receptor agonists, with a dose-response curve and multiple endpoints, will clarify the therapeutic potential of targeting the EP1 receptor for the treatment of ICH. Nevertheless, the main finding that post-treatment with a selective EP1 antagonist aggravates ICH-induced brain injury supports our previous findings suggesting an important role for the EP1 receptor in positively modulating ICH outcomes. Many studies have shown differential effects of modulating PGE_2_-EP1 receptor signaling on outcomes in models of acute and chronic neurologic disorders [10, 11, 14, 15, 24, 28, 31, 32], including contradictory results in the various stroke models. It appears that EP1 receptor agonism and antagonism may be useful under hemorrhagic and ischemic conditions, respectively [11, 24, 33].

Hemolysis following ICH is an important trigger in the progression of brain injury. The liberated free heme generates ferric ions (Fe^3+^), which via the Fenton reaction produce reactive oxygen species, inducing oxidative stress. To understand whether EP1 receptor activation or inhibition modulates ferric iron content, brain sections were subjected to Perl’s iron staining. Analogous to the effects seen with SC-51089 and 17-pt-PGE_2_ treatment on lesion volume, we found an increase in the ferric iron content of antagonist-treated mice, while no significant differences were observed in the agonist-treated group. These data suggest that inhibition of PGE_2_-EP1 receptor signaling prevents the clearance of iron from injured brain regions, which may be a consequence of impaired microglial phagocytosis with EP1 blockade since deletion of this receptor has been reported to reduce phagocytic capability [11]. However, another possible explanation is that antagonist-treated mice have more bleeding, leading to more accumulation of iron in the hemorrhagic area. The latter hypothesis is supported by our previous findings indicating that deletion of the EP1 receptor increases cerebral blood flow [28].

Microglial and astrocyte activation is another criterion to gauge injury severity and monitor the neuroinflammatory response. Following ICH, microglia likely play a dual role where they are protective in their cleanup of red blood cells and damaged brain tissue, thereby promoting repair, but toxic in their facilitation of neuroinflammation and recruitment of leukocytes through the production of proinflammatory cytokines [11, 37]. Here, significantly increased microgliosis was seen in ipsilateral cortical regions with EP1 antagonist treatment, whereas no differences were seen in the ipsilateral striatal regions of the same group or in the agonist-treated group. We have previously shown that the EP1 receptor colocalizes with microglial markers in perihematomal regions and that receptor deletion reduces the activated microglial population and phagocytic function [11]. Several possibilities exist to explain these contradictory striatal microgliosis findings in EP1^−/−^ mice and WT mice treated with an EP1 antagonist: (1) prostaglandin receptors are reported to cross talk [38], and it is possible that EP1 deletion caused the expression of another prostanoid receptor important for regulating microglial activation state to change, (2) deletion of the EP1 receptor reduces basal levels of microglial activation, and (3) EP1^−/−^ mice may develop some other compensatory mechanism. In comparing cortical and striatal post-ICH microglial activation state with EP1 antagonism, cortical microglia appear to retain their ability to activate and respond to the injury, indicating a brain region-specific response.

Astrocytes play diverse roles in response to brain injury, where they try to contain the damage be forming a glial scar [39, 40]. However, excessive or sustained astrocyte activation can be detrimental and augment brain damage and functional deficits by contributing to chronic inflammation and cell death. Treatment with the antagonist had no effect on astrogliosis, whereas the agonist significantly reduced astrogliosis in the ipsilateral cortical and striatal regions. Strong evidence exists suggesting that impairment of astrogliosis improves axonal growth and improves functional outcomes following brain injury [41, 42]. Here, we observed a significant decrease in cortical and striatal astrogliosis in the agonist-treated group, whereas no significant differences were seen with EP1 antagonist treatment. Intriguingly, this reduced astrogliosis with 17-pt-PGE_2_ treatment was accompanied by better neuromuscular functional recovery. The discrepancy in astrocyte and microglial activation with EP1 antagonism or agonism (e.g. agonism decreases astrogliosis, whereas antagonism does not have an effect) may also result from the unbalanced influence of PGE_2_-EP3 receptor signaling between the treatment groups.

Finally, we evaluated leukocyte transendothelial migratory potential, leukocyte infiltration, and BBB disruption, where we found less immunoreactivity for markers of these outcome measures in the agonist-treated group and no significant differences with EP1 antagonism. As previously mentioned in the context of lesion volume, neurobehavioral testing, and gliosis, the interpretation of these data is complicated given the lack of opposing results between the two treatment groups and non-selective nature of the agonist toward EP1/EP3 receptors; although, it does suggest that the EP3 receptor may play a larger role in modulating these particular outcomes when compared to the contribution of PGE_2_-EP1 signaling. ICH initially directly disrupts the BBB, leading to a massive neuroinflammatory cascade that is characterized by invasion of the lesion cite by leukocytes and further damage to the vasculature. There are various triggers that can lead to secondary BBB dysfunction after ICH. Importantly, blood products (e.g. thrombin, hemoglobin, iron) and the inflammatory response, including that mediated by endogenous and infiltrating cells, play a critical role in ICH-induced BBB dysfunction [3, 4]. As previously mentioned, we found that treatment with the EP1 antagonist increased brain ferric iron content, whereas the EP1 agonist had no effect. Higher iron load after ICH means a greater potential for oxidative damage and inflammation, ultimately leading to more BBB damage. However, we did not observe an increase in BBB dysfunction in the antagonist-treated group; instead, we observed improved BBB integrity (as identified by IgG staining) in the agonist-treated mice that did not display less ferric iron content or smaller lesion volumes (i.e. less hemoglobin and thrombin). These findings suggest that in the present study, cell-mediated inflammatory responses may be primarily responsible for the observed differences in BBB integrity between the treatment groups.

Endogenous cell types such as microglia and astrocytes can secrete factors that modulate BBB permeability, especially with excessive or sustained activation. Here, we have shown that agonist-treated mice display significantly reduced activation of one of the key cell types responsible for maintenance of the BBB, astrocytes, and this reduced astrogliosis could be contributing to the overall preservation of BBB integrity with 17-pt-PGE_2_ treatment. However, infiltrating leukocytes such as neutrophils migrate to the site of injury and augment the neuroinflammatory cascade, causing further damage to the BBB. Our findings show that treatment with 17-pt-PGE_2_ decreases neutrophil infiltration, as monitored by immunohistochemical staining for MPO. In the current experimental setting, it is not possible to distinguish the respective contribution of astrogliosis and leukocyte infiltration in modulating BBB integrity, although both processes seem to play a role. Leukocytes cross the endothelium using paracellular and transcellular pathways [43–45]. PECAM-1 is expressed on the surface of several types of leukocytes (including neutrophils) and lateral borders of endothelial cells, and is involved in the transmigration phase of leukocyte emigration through homophilic interactions [46, 47]. Neutrophil transmigration capability, as tested by PECAM-1 staining, was found to be lower in mice treated with 17-pt-PGE_2_, implying that EP1/EP3 agonism preserves the BBB barrier and reduces leukocyte infiltration by sealing intercellular gaps between endothelial cells, and probably more importantly, decreasing the engagement of migrating leukocytes.

## Conclusions

In this study we have provided additional evidence consistent with our previous findings suggesting a neuroprotective role for the EP1 receptor following ICH. Our results show that blockade of the PGE_2_-EP1 signaling axis aggravates ICH-induced brain injury and increases ferric iron deposition and microgliosis, whereas stimulation decreases astrogliosis, leukocyte transendothelial migratory potential, neutrophil infiltration, and BBB breakdown and improves neuromuscular functional recovery. Caution should be used when interpreting and extrapolating these results because it is likely that a portion of the findings are due to activation of EP3 signaling rather than EP1, given the non-selective nature of the agonist used in this study. Even so, EP1-mediated post-ICH neuroprotection is probably achieved by positive modulation of microglial phagocytosis and/or decreased bleeding tendency, leading to lower iron overload, less oxidative processes, and attenuated neuroinflammatory responses. Additional studies with other ICH models, a selective EP1 agonist, and pharmacologic manipulation of the EP3 receptor will clarify the respective role of these PGE_2_-mediated signaling axes after ICH. Nonetheless, the EP1 receptor remains a viable therapeutic target for the treatment of ICH.

## Abbreviations

ABC: avidin/biotin enzyme complex
AD: Alzheimer’s disease
BBB: blood brain barrier
cAMP: cyclic adenosine monophosphate
COX: cyclooxygenase
DAB: 3,3′-diaminobenzidine
DMSO: dimethyl sulfoxide
EP1: E prostanoid receptor subtype 1
EP3: E prostanoid receptor subtype 3
EP1^-/−^: EP1 receptor knockout
GFAP: glial fibrillary acidic protein
HIE: hypoxic-ischemic encephalopathy
Iba1: ionized calcium-binding adapter protein 1
ICH: intracerebral hemorrhage
IgG: immunoglobulin G
mPGES: membrane-bound PGE synthase
MPO: myeloperoxidase
PBS: phosphate-buffered saline
PD: Parkinson’s disease
PECAM-1: platelet endothelial cell adhesion molecule 1
PFA: paraformaldehyde
PGE_2_: prostaglandin E_2_
SEM: standard error of the mean
TBI: traumatic brain injury
WT: wildtype
